# ontologyX: a suite of R packages for working with ontological data

**DOI:** 10.1093/bioinformatics/btw763

**Published:** 2016-12-30

**Authors:** Daniel Greene, Sylvia Richardson, Ernest Turro

**Affiliations:** 1 MRC Biostatistics Unit, Cambridge Biomedical Campus, Cambridge Institute of Public Health, Cambridge, UK; 2 Department of Haematology, University of Cambridge School of Clinical Medicine, Cambridge Institute for Medical Research, Wellcome Trust/MRC Building, Cambridge, UK

## Abstract

**Summary:**

Ontologies are widely used constructs for encoding and analyzing biomedical data, but the absence of simple and consistent tools has made exploratory and systematic analysis of such data unnecessarily difficult. Here we present three packages which aim to simplify such procedures. The ontologyIndex package enables arbitrary ontologies to be read into R, supports representation of ontological objects by native R types, and provides a parsimonius set of performant functions for querying ontologies. ontologySimilarity and ontologyPlot extend ontologyIndex with functionality for straightforward visualization and semantic similarity calculations, including statistical routines.

**Availability and Implementation:**

ontologyIndex, ontologyPlot and ontologySimilarity are all available on the Comprehensive R Archive Network website under https://cran.r-project.org/web/packages/.

**Supplementary information:**

Supplementary data are available at *Bioinformatics* online.

## 1 Introduction

Ontological annotation is now used to describe many different biological phenomena, including gene function (Gene Ontology Consortium *et al.*, 2015) and human phenotype abnormality (Köhler *et al.*, 2014), with many ontologies, and ontological datasets publicly available. Accounting for dependency between terms induced by the structure of their ontologies is vital for downstream statistical analysis and visualization. Therefore, software methods are required which integrate ontologies and ontological data with mainstream statistical programming environments, so that the data can be analyzed effectively. The ontoCAT (Adamusiak *et al.*, 2011) package enables simple querying and traversal of ontologies, but many of its key functions are slow and it requires a Java runtime installation.

There are software packages enabling manipulation and plotting of graphs, for example graph (Gentleman *et al.*, 2016) and Rgraphviz (Hansen *et al.*, 2016) respectively, which can be used to view sections of ontologies. However, their functions are low level, which makes procedures such as plotting of ontological term sets and fine-grained control of graphical parameters quite involved. There are R packages which provide procedures for computing semantic similarities between terms and sets of terms for specific ontologies (Fröhlich *et al.*, 2007; Yu *et al.*, 2010, 2015) but they do not support semantic similarity computation for arbitrary ontologies. Furthermore, the currently available methods are too slow to apply to large datasets.

Here we present a suite of R packages, dubbed ‘ontologyX’, consisting of ontologyIndex, ontologyPlot and ontologySimilarity, which together address the issues described and form a consistent interoperable set of tools that is readily extensible with additional ontological functionality.

## 2 Methods

**Fig. 1:**
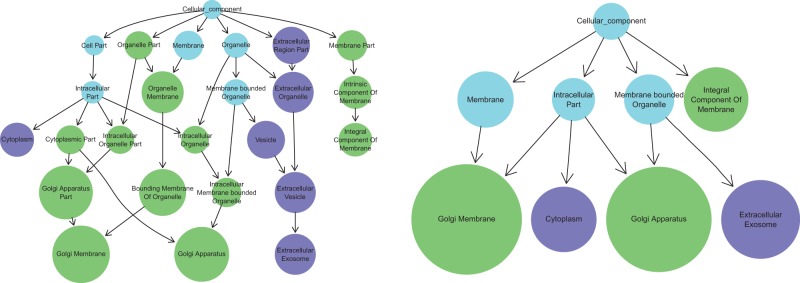
Plot of terms descending from the cellular_component term in the GO, extracted using the exclude_descendants function from ontologyIndex, for genes *QPCTL* and *CRNN* using ontologyPlot. The left panel shows the full set of ancestral terms used in the annotation of the genes, while the right panel shows only those remaining after remove_uninformative_terms has been called. Terms annotated to both genes, either implicitly or explicitly, are shown in light blue, while those annotated only *QPCTL* and *CRNN* are shown in green and purple respectively. The size of the nodes has been set to be proportional to the information content (i.e. negative log frequency) of the terms with respect to gene annotation downloaded from the GO website

ontologyIndex is an R package which was developed in order to provide a terse, low-level and easy to use set of functions for exploiting the structure of ontologies. Ontologies can be read into R from files in Open Biomedical Ontologies (OBO) format, with most commonly used ontologies available in this format on the OBO Foundry’s website (Smith *et al.*, 2007) . Ontologies which are only available in a Web Ontology Language (OWL) format may be used by first converting them into OBO format, for example using the ROBOT command line tool (Overton *et al.*, 2015) . A custom internal representation of ontologies—the ontology_index class—is used which stores properties of terms including term ancestors, enables fast ontological operations, and can be queried using base R functions. It uses native R types to represent ontological terms and sets of ontological terms, enabling simple integration with R’s features, high-level functions and other packages. It includes functions for performing set operations respecting the structure of the ontology, for example: exclude_ descendants, which given term sets *A* and *B*, excludes terms in *B* and their descendants from set *A*; prune_descendants, which preserves terms in *B* which are ancestors of terms in *A* after applying exclude_descendants), and minimal_set, which maps a set of ontological terms onto a non-redundant set. ontologyIndex is lightweight, fast (see Table 1) and readily extended by other packages. For example, the R package gsEasy (Greene, 2016) facilitates gene-set enrichment analysis (Subramanian *et al.*, 2005) using the get_ancestors function to propagate parent-child relations through the GO. ontologyPlot extends ontologyIndex with functions which considerably ease the task of plotting sets of ontological terms and the ‘is-a’ relations between them, as the user need only pass an ontology_index and a vector of term IDs to the plotting function. It includes several functions for transforming sets of terms to distill the important features for particular visualizations. For example, given a set of ontologically annotated objects, the function remove_uninformative_terms removes terms whose children are annotated to the same objects, leading to simpler diagrams. Figure 1 demonstrates how ontologyPlot can be used to visualize GO annotation for *QPCTL* and *CRNN*, and the effect of using remove_uninformative_terms to simplify the figure. ontologyPlot utilizes the Rgraphviz package’s interface to the graphviz (Gansner and North, 2000) graphical layout engine. It further allows graphs to be exported in standard DOT format and does not constrain the graphical parameters, so users can take full advantage of options in any rendering software.

**Table 1:** Mean execution time for retrieving descendants and ancestors for individual terms in the Human Phenotype Ontology

	Descendants (ms)	Ancestors (ms)
ontoCAT	11.99	12.75
ontologyIndex	0.38	0.14

Semantic similarity quantifies similarity between ontological terms and sets of ontological terms. ontologySimilarity extends ontologyIndex to enable similarities between ontological objects to be computed given an ontology_index and sets of term IDs. It facilitates the calculation of similarity at three levels: between ontological terms (ID strings), between ontologically annotated objects (ID string vectors), and within groups of ontologically annotated objects (lists of ID string vectors). It implements Resnik’s (Resnik *et al.*, 1999) and Lin’s (Lin, 1998) expressions for the similarity of terms. Unlike other packages for calculating semantic similarities, ontologySimilarity does not depend on static, pre-built SQLite databases or Bioconductor annotation packages and works with arbitrary term annotations. Furthermore, it offers inferential procedures such as get_sim_p, which assesses the strength of similarity between groups of objects (Westbury *et al.*, 2015) . Flexible functions facilitate use in complex methods, for example as in the R package SimReg (Greene *et al.*, 2016), which implements a semantic similarity based regression algorithm. All similarity routines are written in C ++ and called from R (Eddelbuettel *et al.*, 2011), and the user can balance performance and memory usage for downstream analysis by selecting whether to store similarities between terms or term sets, or store an index for fast similarity lookups. We compared the performance of ontologySimilarity against other packages offering functions for calculating pairwise term and gene similarities, the results of which are shown in Table 2. The results indicate that ontologySimilarity executes substantially faster, and suggests tangible advantages for use with large datasets.

**Table 2:** Execution times for computing pairwise similarity matrices for 1000 randomly selected GO terms and 100 randomly selected gene GO annotation sets using Lin's expression for term similarity

	Term sim (s)	Gene sim (s)
GOSim	1075.43	298.34
GOSemSim	1.71	116.72
ontologySimilarity	0.31	0.06
ontologySimilarity (indexed)		0.04

## 3 Conclusion

The key advantage of ontologyIndex is that it can read in arbitrary ontologies, integrates naturally with R, and provides a solid base for extension. ontologyPlot enables uniquely simple and aesthetically pleasing visualization of ontological terms and ontological annotation with a wide variety of graphical options. ontologySimilarity facilitates fast and flexible semantic similarity functionality for ontological objects including assessment of statistical significance and is suitable for application to high-throughput datasets.

*Software*: The following versions of software packages were used to generate the results presented in this manuscript: ontologyIndex 2.2, ontologyPlot 1.4, ontologySimilarity 2.1, GOSim 1.11, GOSemSim 1.99.4 and ontoCAT 1.26.0.

## Funding

This work was supported by National Institute for Health Research award RG65966 (D.G. and E.T.) and the Medical Research Council programme grant MC_UP_ 0801/1 (D.G. and S.R.).

*Conflict of Interest*: none declared.

## Supplementary Material

Supplementary Data
