# Supplementary material for: ontologyX: a suite of R packages for working with ontological data
Source: Bioinformatics. 2016 Dec 30;33(7):1104–6. doi: 10.1093/bioinformatics/btw763 (PMC5386138; doi:10.1093/bioinformatics/btw763)
Supplement: Supplementary Data [file btw763_supp.pdf]

Code used to generate tables and figures

The following versions of software packages were used to generate the results presented in this manuscript: ontologyIndex 2.2, ontologyPlot 1.4, ontologySimilarity 2.2, GOSim 1.11, GOSemSim 1.99.4 and ontoCAT 1.26.0.

Comparing performance of typical ontological queries with **ontoCAT**

```
library(ontoCAT)
library(ontologyIndex)

set.seed(1)

if (!exists("hpo_file_path")) stop("Download HPO from: 'http://purl.obolibrary.org/
   obo/hp.obo' and set 'hpo_file_path' variable to its path")
#original computation based on HPO data-version: releases/2016-04-01

hpo_ontologyIndex <- get_OBO(hpo_file_path)
hpo_ontoCAT <- getOntology(hpo_file_path)

ancestors <- expression(
        ontoCAT=ontoCAT::getAllTermParentsById(object=hpo_ontoCAT, id=term),
        ontologyIndex=ontologyIndex::get_ancestors(hpo_ontologyIndex, terms=term)
)

descendants <- expression(
        ontoCAT=ontoCAT::getAllTermChildrenById(object=hpo_ontoCAT, id=term),
        ontologyIndex=ontologyIndex::get_descendants(hpo_ontologyIndex, roots=term)
)

n_terms <- length(hpo_ontologyIndex$id)

desc_times <- lapply(descendants, function(x) { system.time(sapply(
   hpo_ontologyIndex$id, function(term) eval(x))) })
anc_times <- lapply(ancestors, function(x) { system.time(sapply(hpo_ontologyIndex$
   id, function(term) eval(x))) })

#print results table
print(data.frame(check.names=FALSE, stringsAsFactors=FALSE, `Descendants (ms)`=
   sapply(desc_times, function(x) 1000*as.numeric(x["elapsed"])/n_terms), `
   Ancestors (ms)`=sapply(anc_times, function(x) 1000*as.numeric(x["elapsed"])/
   n_terms)))
```

Using ontologyPlot to visualise QPCTL and CRNN GO annotation

```
library(ontologyIndex)
library(ontologyPlot)
library(ontologySimilarity)
data(go)
data(gene_GO_terms)
data(GO_IC)

cc <- go$id[go$name == "cellular_component"]
cc_anno <- lapply(gene_GO_terms[c("QPCTL","CRNN")],
        function(x) get_ancestors(go, intersection_with_descendants(go, roots=cc, x
            )))

all_terms <- unique(unlist(use.names=FALSE, cc_anno))
QPCTL <- cc_anno$QPCTL
CRNN <- cc_anno$CRNN

terms <- remove_uninformative_terms(go, list(QPCTL, CRNN))

pdf(file="GO-plot.pdf", width=10.5, height=4)
par(mfrow=c(1, 2))
onto_plot(go, terms=all_terms,
        width=calibrate_sizes(GO_IC[all_terms], low=1, high=3),
        fontsize=50,
        fillcolor=rgb(0, all_terms %in% QPCTL, all_terms %in% CRNN, 0.5))

onto_plot(go, terms=terms,
        width=calibrate_sizes(GO_IC[terms], low=1, high=3),
        fillcolor=rgb(0, terms %in% QPCTL, terms %in% CRNN, 0.5))
dev.off()
```

Comparing performance of semantic similarity computation

```
library(ontologyIndex)
library(ontologySimilarity)
library(GOSemSim)
library(GOSim)
set.seed(1)

initialise <- expression(
        ontologySimilarity={ data(go); IC_os <- descendants_IC(go) },
        GOSim=GOSim:::initialize(),
        GOSemSim=hsGO <- godata('org.Hs.eg.db', keytype = "SYMBOL", ont="MF",
           computeIC=TRUE)
)

# GOSim uses Entrez Gene IDs which we need to map to HGNC symbols for comparison
   with GOSemSim and ontologySimilarity. We obtained the mapping using the table
   downloaded from the HUGO Gene Nomenclature Committee website, using the custom
   download page (http://www.genenames.org/cgi-bin/download) and selecting the '
   Approved Symbol' and 'Entrez Gene ID' columns, and saving it as 'symbol.txt' in
   the working directory.
stopifnot(file.exists("symbol.txt"))
symbol_map <- read.table(file="symbol.txt", header=TRUE, sep="\t", comment.char="",
     stringsAsFactors=FALSE)

env <- environment()

ini_times <- lapply(initialise, function(x) { cat(as.character(x), "\n");
   system.time(eval(x, envir=env)) })

IC <- slot(hsGO, "IC")

gene_anno <- with(data=slot(hsGO, "geneAnno"), split(f=SYMBOL, x=GO))

n_genes <- 100
n_terms <- 1000

genes <- names(gene_anno)[1:n_genes]

terms <- names(which(IC < Inf & IC > 0))[1:n_terms]

#create expressions for calculation of term/gene similarity matrices for comparison
#note that different values are obtained in the matrices as different sources of
   information content are used.

term_similarity_matrix <- expression(
        GOSim=getTermSim(terms, method="Lin", verbose=FALSE),
        GOSemSim=mgoSim(terms, terms, semData=hsGO, measure="Lin", combine=NULL),
        #ontologySimilarity uses Lin's expression for term similarity by default
        ontologySimilarity=get_term_sim_mat(go, information_content=IC_os[
           get_ancestors(go, terms)], row_terms=terms, col_terms=terms),
        `ontologySimilarity (indexed)`=NA
)

gene_similarity_matrix <- expression(
        GOSim=getGeneSim(as.character(na.omit(symbol_map$Entrez.Gene.ID[match(genes
```

```
            , symbol_map$Approved.Symbol)])),similarity="funSimMax",similarityTerm="
            Lin",verbose=FALSE),
        GOSemSim=mgeneSim(genes, semData=hsGO, measure="Lin", combine="BMA",
            verbose=FALSE),
        ontologySimilarity=get_sim_grid(ontology=go, information_content=IC_os,
            term_sets=gene_anno[genes]),
        `ontologySimilarity (indexed)`=local({ term_sim_mat <- get_term_sim_mat(go,
             information_content=IC_os[get_ancestors(go, unique(unlist(use.names=
            FALSE, gene_anno[genes])))]); get_sim_grid(term_sim_mat=term_sim_mat,
            term_sets=gene_anno[genes]) })
)

calc_times <- lapply(list(term_sim=term_similarity_matrix, gene_sim=
    gene_similarity_matrix), function(calc_type) lapply(calc_type, function(x) if (
    is.na(x)) NA else system.time(eval(x))))

#print results table
print(data.frame(check.names=FALSE, stringsAsFactors=FALSE, `Term sim (s)`=sapply(
    calc_times$term_sim, function(x) as.numeric(x["elapsed"])), `Gene sim (s)`=
    sapply(calc_times$gene_sim, function(x) as.numeric(x["elapsed"]))))
```
